# Feasibility of a group-based intervention to enhance health-related quality of life and physical activity in children and adolescents with chronic illness: a study protocol

**DOI:** 10.1186/s40814-025-01682-w

**Published:** 2025-07-17

**Authors:** Lars Peder Vatshelle Bovim, Elisabeth Ørskov Rotevatn, Haakon Kristian Kvidaland, Bård Bogen, Thomas Halvorsen, Mette Engan

**Affiliations:** 1 Children and Youth Clinic, Haukeland University Hospital, Bergen, Norway; 2 Department of Clinical Science, Faculty of Medicine, University of Bergen, Bergen, Norway; 3 Department of Global Public Health and Primary Care, Faculty of Medicine, University of Bergen, Bergen, Norway; 4 Department of Sports Medicine, Norwegian School of Sport Sciences, Oslo, Norway

**Keywords:** Chronic illness, Chronic diseases, Children, Adolescents, Health-related quality of life, Physical activity, Complex Intervention

## Abstract

**Background:**

Chronic or long-term illnesses in early years increase the risk of comorbidities such as mental disorders, social exclusion, and difficulties in forming relationships. In Bergen, Norway, the three-phased intervention Life Coping Program is established. This program is designed to support adolescents with chronic or long-term illnesses through tailored preparation, a hospital-based Life Coping Course, and structured follow-up aimed at empowering them to actively manage their health and adopt a more active lifestyle. This protocol paper describes a feasibility trial of the Life Coping Program, focused on improved health-related quality of life (HRQoL) and levels of physical activity.

**Methods/design:**

This study is based on the British Medical Research Council’s (MRC) modified framework for developing and evaluating complex interventions. Feasibility and acceptability of the Life Coping Program will be evaluated in an uncontrolled open-label trial using quantitative measures. Sixty children and adolescents with chronic illness or severe medical conditions will be recruited along with one associated caregiver. Feasibility will be assessed by tracking attendance and participation in the different parts of the intervention. Acceptability of the intervention will be assessed by patient-reported questionnaires. HRQoL and levels of physical activity will be measured using the KidScreen-27 and Actigraph GT3X + monitor, respectively, at baseline and 1 and 6 months post-intervention.

**Discussion:**

The study will explore the feasibility of an innovative treatment strategy targeting children and adolescents at risk of poor health-related outcomes across multiple dimensions. The findings will inform the design of a future randomized controlled trial.

Trial registration.

ClinicalTrials.gov, NCT06709248. Registered 15th of November 2024—retrospectively registered, https://clinicaltrials.gov/study/NCT06709248?term=NCT06709248&rank=1

## Background

During the recent decades, medical advancements have improved treatment options for life-threatening and chronic diseases, resulting in an increasing number of children and adolescents surviving severe illnesses. However, many of these children and adolescents grow up with a variety of long-term health problems and sequelae, relating both to their conditions and their treatments. Living with chronic or long-term illnesses has been shown to increase the risk of comorbidities and various challenges, including mental disorders, social exclusion, and difficulties in forming relationships [1]. Prolonged illness during the formative years of childhood and adolescence is also associated with fewer opportunities or motivation to engage in social and physical activities [2–6] and with reduced health-related quality of life (HRQoL), both in childhood and onwards into adult life [2]. These associations have been found across a wide variety of pediatric conditions including survivors of childhood cancer [6, 7], children born prematurely [8], chronic pain [9], congenital heart defects [10], severe burn injuries [11, 12] and for children with oral cleft [13]. However, findings are not universally consistent, as some studies have reported no reduction of HRQoL when compared to peers [14]. This underscores the variation of individual outcomes and highlights a need for a tailored follow-up.

Physical activity has been shown to be important for children and adolescents, both as a social arena and for the development of social and physical skills and capacities [15, 16]. Typically, primary school children are more physically active than adolescents, both with and without disabilities [17]. Efforts to increase physical activity during childhood are believed to be important for developing good exercise habits that can sustain a good health throughout life [18]. In addition to the prevention of lifestyle diseases [19, 20], engaging in physical activities provides both psychological and social health benefits, prevents mental health problems, enhances self-confidence, and improves social interaction skills [21–26].

Several factors influence participation in physical activity, including perceived physical competence [21], parental support [27], and access to sports activities [16, 21, 27]. It is important to understand these barriers and opportunities, their underlying drivers, and their origins. Interventions aiming to increase participation in physical activity have shown a positive impact on activity level and HRQoL [28, 29]. However, Martin Ginis et al. [27] identified over 200 factors related to physical activity participation among children and adults with physical disabilities; hence, interventions are often complex as they aim to address various aspects of this broad spectrum of contributing factors. Despite the diverse nature of severe illnesses during childhood, many of these patients may share several common issues. Healthcare systems should provide education on long-term coping strategies to increase HRQoL and motivation to be physically active. Involving both parents and the local community may be beneficial when empowering and supporting chronically ill children and adolescents [30, 31].

The intervention “Life Coping Program” was developed at the Children and Youth Clinic, Haukeland University Hospital, Bergen, Norway, during 2020–2023. The clinic serves as a local hospital for approximately 100,000 children and adolescents, in addition to providing several regional and national functions. In 2023, the clinic had around 4000 admissions and 33,000 outpatient consultations [32]. Following two workshops in 2020 and 2021 that gathered clinicians and researchers from several sections of the clinic, and inputs from parents of patients and two youth representatives, a clinical team of three responsible health care professionals was established. This team, in cooperation with researchers, clinicians, administrators, and leaders, identified and developed necessary organizational infrastructure, work procedures, and a research protocol with a framework for data collection that was practical for both clinical and scientific use.

The overarching goal of the Life Coping Program intervention is to empower children, adolescents, and their families to actively manage their health challenges and to inspire and support young individuals in adopting and sustaining an active lifestyle while reducing sedentary behavior. The specific goals include enhancing HRQoL and physical activity levels in children with chronic illnesses or medical conditions, as well as decreasing the need for specialized healthcare follow-ups.

This study protocol describes a feasibility trial of the group-based intervention Life Coping Program for children and adolescents living with chronic illnesses or being survivors of severe medical conditions.

The aims of the trial are as follows:

1. To quantify fidelity and willingness to participate, as well as attendance and retention rates.
2. To assess the acceptability and practicality of assessment tools and the intervention.
3. To provide power estimations of effectiveness of the intervention on HRQoL and physical activity levels in a real-life hospital setting.

## Method and design

The planned study is structured as an uncontrolled, open-label feasibility trial using a pre-test–post-test within-subject design, based on ongoing clinical practice. The study design is based on the British Medical Research Council’s (MRC) framework [33, 34].

Our primary outcome measure will be the proportion of completed elements of the intervention, with patient-specific outcome measures as secondary outcomes. The findings will provide an indication of whether the ongoing clinical practice is suitable for a wide aspect of primary conditions in a hospital setting. Additionally, the results from this study will help identify necessary adjustments to the Life Coping Program and evaluation methods, informing future phases of evaluation and implementation.

**Fig. 1:**
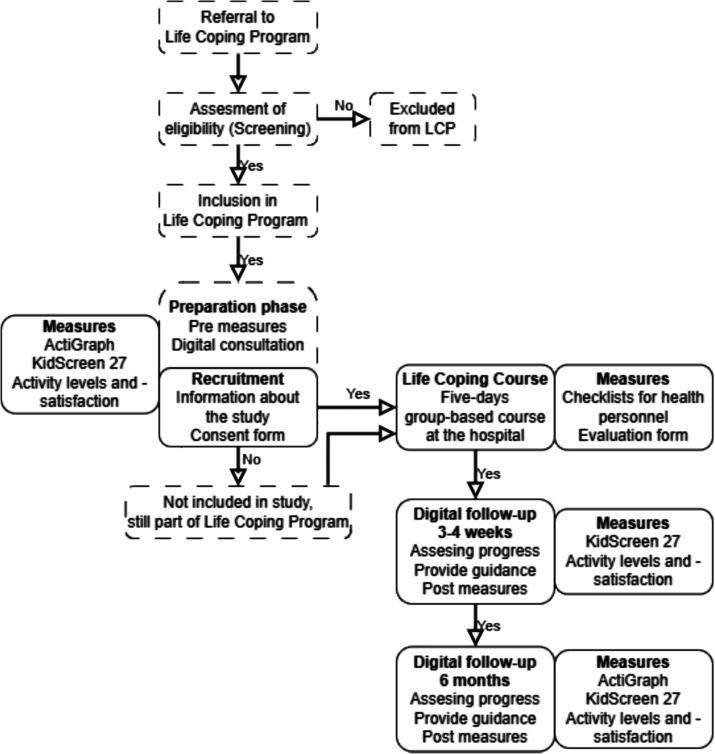
Study flow chart for Life Coping Program feasibility study. Dashed line for only clinical activities, and bold line for activities included in the study. LCP Life Coping Program

The overall study design is depicted in Fig. 1. Details of enrollment, interventions, and assessments are described in Table 1.

**Table 1.**
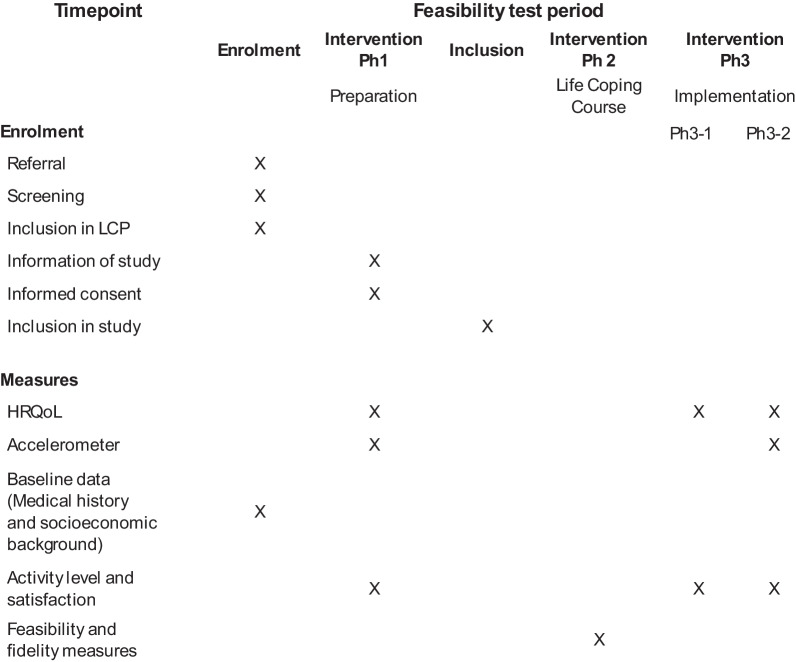
SPIRIT table for an uncontrolled open-label feasibility testing of a group-based intervention: Life Coping Program

*Ph* Phase, *Ph3-1 *Follow-up 3-5 weeks after the Life Coping Course, *Ph3-2* Follow-up 6 months after the Life Coping Course, *HrQoL* Heath related Quality of Life

### Feasibility, fidelity, and acceptability

In complex intervention research, a wide range of methods and measures might be relevant, depending on the intervention itself, the phase in which the key uncertainties are best evaluated, and the priorities of the stakeholders [34]. In a feasibility trial, the aim is to assess feasibility and acceptability of an intervention and evaluation measures to stall, adjust, or progress to the next stage of evaluation, for example effect studies. Whereas *feasibility* refers to the extent to which an intervention is successfully delivered as intended, including recruitment, retention, resources used et cetera, *fidelity* focuses on the quality of the implementation, covering whether the intervention is delivered as planned and anticipated [34]. *Acceptability* is inadequately defined in the literature but can be understood as the extent to which the target population and those delivering the intervention consider it appropriate or satisfactory, and can be measured through behavior (e.g., retention), affect (e.g., feelings) or cognition (e.g., reflections) [35].

### Participants and recruitment

Patients for the Life Coping Program are identified through clinical practice at Haukeland University Hospital, Bergen, Norway. Clinicians, including physicians, nurses, and physiotherapists, working with patients with relevant primary conditions, are eligible to refer them to the Life Coping Program (Table 1). Referrals are then evaluated by a representative from the clinical team and an attending physician from the relevant field (screening).

*Eligible participants for the study* are patients aged 9 to 16 years who are attending the Life Coping Program for one of the following primary conditions: congenital heart defects, congenital oral clefts, premature birth before 32 weeks of gestation, completed cancer treatment, chronic pain conditions, or significant burn injuries requiring treatment and follow-up care. Both the patient and one caretaker must be able to answer questionnaires in the Norwegian language. As part of the preparation (phase 1), potential participants are invited to be included in the study, both in writing and verbally, following templates from the Regional Committees for Medical and Health Research Ethics. Signed consent is collected. Given that the Life Coping Program is embedded in standard clinical practice, all eligible patients receive identical treatment and follow-up, regardless of their consent to participate in the study (Fig. 1). Participants can withdraw their consent at any time without reason, as explained in the consent forms. Withdrawal from the study will not impact future participation in the Life Coping Program or other benefits. All data not used in analyses and publications will then be removed from the study server.

Eligible participants are excluded if they have a medical condition or treatment plan that, as determined by the healthcare professional or project staff, may hinder or alter study participation. Examples include a deteriorating medical condition requiring hospitalization or elective surgery that is scheduled during the Life Coping Course.

### Therapists

The clinical team comprises health professionals with established knowledge of physical activity, youth development, and group dynamics. These core clinicians, referred to as *Primary Contacts*, may include physiotherapists, nurses, occupational therapists, or exercise physiologists, but no more than three in each team, depending on availability and the patients’ needs. The clinical team for each particular group is complemented by a medical specialist and a nurse with expertise in the relevant primary condition, along with representatives from relevant patient organizations.

### Intervention—the Life Coping Program

**Fig. 2:**
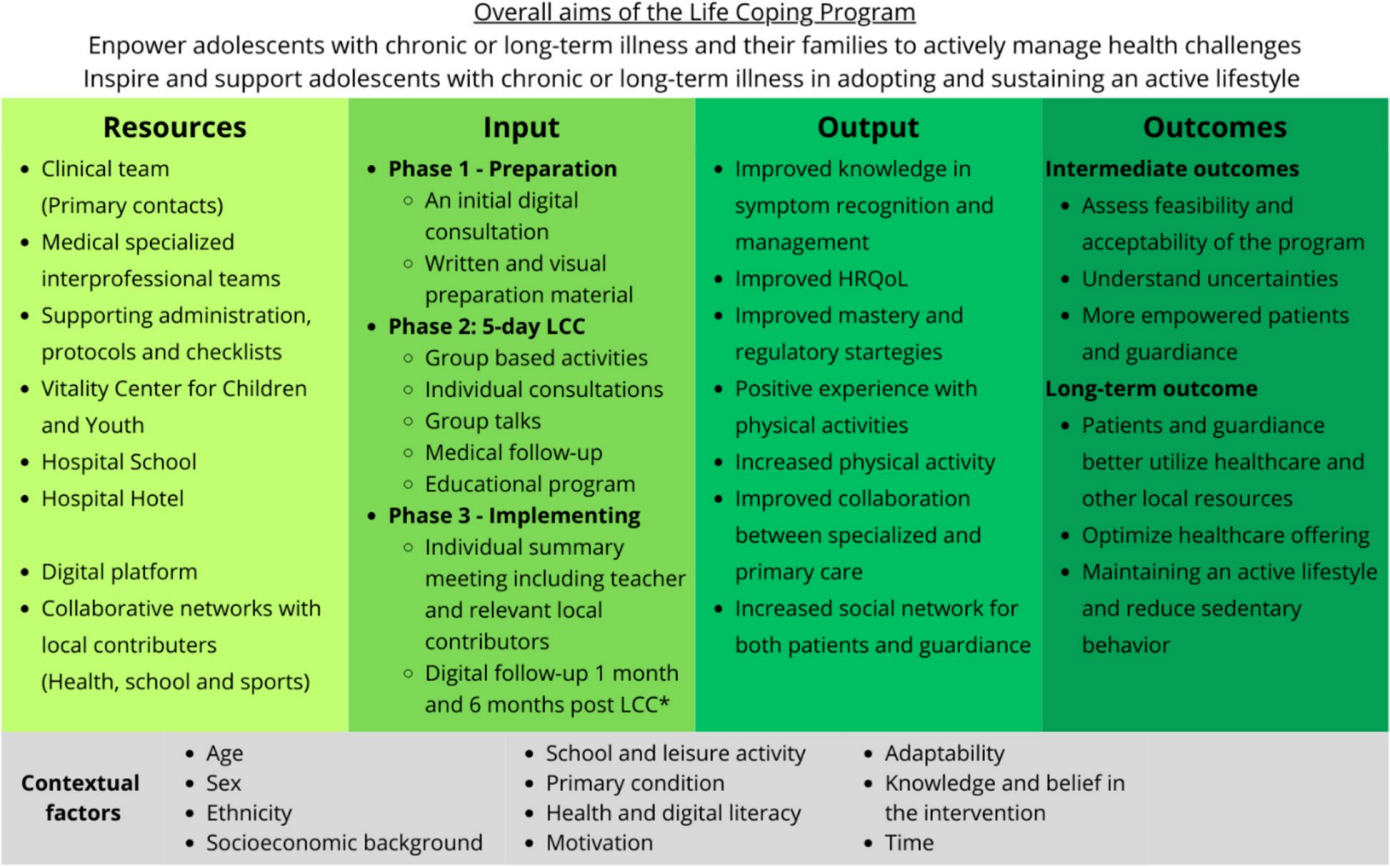
The logic model for the Life Coping Program presents the aim, resources, input and anticipated output and outcomes of the intervention. Anticipated contextual factors are also presented. A logical model is dynamic, and updated versions can be found on clinicaltrials.gov, NCT06709248. LCC Life Coping Course, HRQoL health-related quality of life

Patients included in the established Life Coping Program are grouped by their primary condition, meaning the diagnosis or condition prompting the referral. Four to eight families are matched per group, with a preferred target of six. Each family is assigned a dedicated Primary Contact throughout the program. The Life Coping Program consists of three phases: (1) preparation, (2) life coping course, and (3) implementation at home. Theoretical and logical rationale for the intervention is presented in the logic model (Fig. 2).

#### Phase 1: preparation

*Written and visual preparation materials* are presented, aiming to motivate and inform the adolescent, caregivers, and potential siblings and friends.

*Questionnaires* regarding personal resources, activity levels, coping skills, and social relationships (KidScreen-27 [36] and purpose-made questionnaires) are collected and analyzed by the healthcare professional as preliminary information before the primary consultation.

*A primary digital consultation* is conducted 3 to 5 weeks before the Life Coping Course. Additional to practicalities, the following topics are discussed and explored with the patient and caregiver:

- Aims—improving quality of life in chronic illness
- Setting and reaching goals—during and after the Life Coping Program
- Commitment—participation only if ready and willing
- Follow-up—Establishing framework for the implementation phase at home (including team for Phase 3)

#### Phase 2: Life Coping Course

The Life Coping Course is a 5-day stay at the hospital, from Monday to Friday. The children or adolescents and one caretaker reside at the hospital patient hotel while all activities take place at two specific locations at the Haukeland University Hospital: the Vitality Center for Children and Youth, and at the hospital school. The objectives of the course are to educate and to foster a sense of mastery and camaraderie among individuals and families with common illnesses or medical conditions. Participants are to be equipped with strategies to effectively cope with symptoms and discomfort in their daily activities, ultimately aiming to enhance their sense of security, overall well-being, and HRQoL in both the short and long term.

The generic schedule for the Life Coping Course includes the following:

- *Individual consultations* Monday and Friday (see phase 3)
- *Group-based physical activities* with tailored goals and focus areas
- *School activities in groups* emphasizing life skills and creating lasting memories
- *Excursion* to experience mastery, enjoyment, and face challenges outside the home
- *Social activity* organized by patient organizations to build network
- *Group talks* for sharing experiences and strategies, adolescents and parents separately.
- *Medical follow-up* at specialist outpatient clinic, if deemed relevant by the medical team
- *Parental educational program* (e.g., social worker, nutritionist)
- *Social activities* (cinema, gaming, and activity courses) for mastery and social development
- *Communal meals* to foster social skills and networking

#### Phase 3: implementing changes at home

The implementation phase of the Life Coping Program consists of the following:

*An individual summary meeting* is conducted on the final day of the Life Coping Course, in a hybrid format (in-person and digital participation). Participants included in this meeting are the child or adolescent, both their parents/caretakers, primary contact, and any relevant individuals agreed upon in phase 1, such as the physical education teacher, an Activity Guide (a facilitator for inclusion in organised sports activities) [37], a coach from the local sports club or a significant person in the family's life, the primary care physician and/or school health services. The participation of all the individuals in this summary meeting is agreed upon by both the adolescent and the caregivers. The objectives of the summary meeting are to evaluate the progress and achievements of the week and to establish a framework for the ongoing implementation of what the participant and family have learned and the goals they have set during the week.

*Digital follow-up consultation* 3–4 weeks after the Life Coping Course, assessing the progress made towards achieving the goals and providing guidance on any challenges that may hinder goal attainment.

*Digital follow-up consultation* 6 months after the Life Coping Course, for establishing a long-term commitment and assessing the long-term progress and achievement of set goals (Fig. 1).

### Data collection instruments

A wide range of data collection instruments are planned to be used in this trial. Checklists and evaluation forms are specifically for the feasibility stage, whilst instruments for HRQoL and subjective and objective activity levels are part of the intervention.

*Checklists for health personnel* are developed and to be filled out after six defined sessions of the program: the Primary digital consultation in phase 1, primary individual consultation and Activities during the Life Coping Course in phase 2, and Individual summary meeting and digital follow-up consultation 1 month and 6 months after in phase 3. The checklists consist of practical elements (attendance, clarifications) and predefined key elements of each session. They are to be answered with yes/no per element, and a comment if no is chosen (Appendix).

An *Evaluation form for the Life Coping Course* aims to assess adolescents and caregivers’ experiences and acceptance of the 5-day course. The questionnaire covers practical aspects, perceived relevance of the activities, interactions with healthcare staff, and overall alignment with their life situation.

*HRQoL* will be measured using the standardized questionnaire KidScreen-27 [36]. The KidScreen-27 is a widely used instrument designed to assess HRQoL in children and adolescents. It evaluates five key dimensions: physical well-being, psychological well-being, autonomy and parents, social support and peers, and school environment. The Norwegian version of the KidScreen-27 has demonstrated good psychometric properties: Cronbach’s alpha values for internal consistency ranged from 0.73 to 0.83 across the five domains, and the test–retest reliability described by interclass correlation coefficient was in the range of 0.71 to 0.81 in a study in Norwegian schoolchildren (38). In the current study, both the parent version and a child version will be used.

The purpose-built questionnaires *Self-reported activity levels and satisfaction with relevant activities* and *Parents’ perception of their child’s activity level and satisfaction with relevant activities* will be collected. The questionnaires are partly based on the “Health Behaviour in School-aged Children” (WHO survey) [39].

*Objective physical activity levels* will be assessed using one accelerometer (Actigraph wGT3X-BT, ActiGraph LLC, Pensacola, FL, USA) attached to the prominent hip with an elastic band. Test protocol is set to match the Physical Activity among Norwegian Children Study (PANCS) as described by Hansen et al. [40], ensuring national reference material. Activity recording will take place over 7 days, and the sensor is to be removed only during sleep and water activities (showering and bathing). The accelerometer will be initialized and analyzed using the ActiLife software, version 6. Data will be collected at 30 Hz, and vertical accelerometer data will be derived in epochs of 10 s. Activity levels will be categorized as sedentary (< 100 cpm), low (100–1999 cpm), and moderate-to-vigorous (≥ 2000 cpm) to match potential reference material. See Hansen et al. [40] for further details of the planned analyzing process.

### Outcome measures

The primary outcome measure will be the *number of participants who have completed all elements of the Life Coping Program*: checklists for health personnel, questionnaires for participants, and objective measures of physical activity levels.

The secondary outcomes are as follows:

*Paper-and-pencil versus digitally based questionnaires*. There is conflicting evidence on adherence to the use of paper-and-pencil versus digitally based questionnaires, with indications of paper-and-pencil having higher response rates, and digitally based questionnaires having superior cost-effectiveness and completeness of data [41]. Preparation materials and questionnaires are therefore presented analogously (handouts and paper-and pencil) for the first half of the participants and digital (internet based) for the last half of the included participants in order to explore which format is more feasible in this particular setting.

Adolescents and caregivers’ *experience and acceptance of the 5-day course* Life Coping Course.

*Baseline and changes from pre- to post-intervention* in HRQoL, self-reported activity levels, and objective measured activity levels as measured at baseline, 1 month post- and 6 months post Life Coping Course.

### Statistics

Primary outcome will be presented as percentages (%) with 95% confidence interval (CI). Completion rate will be presented for total treatment completion (completed 6-month follow-up) and for each phase of the intervention. Descriptive data will be presented as means and 95% CI for continuous variables, and as counts (*n*) and percentages (%) and 95% CI for categorical variables. The current study is underpowered to detect statistically significant changes, but within-group analyses will be made for calculating power and sample sizes for forthcoming trials.

Comparison of paper-and-pencil and digital questionnaires will be evaluated, and the two will be presented separately only if conflicting adherence is found. Changes in HRQoL will be assessed based on KidScreen-27 scores, between pre-measures before the Life Coping Course and post measures 3 to 4 weeks and 6 months after. Changes in physical activity will be evaluated based on differences in time spent in sedentary and moderate-to-vigorous intensity levels, between pre-measures before the Life Coping Course and post measures 6 months after.

### Sample size justification

A formal sample size calculation is not required for pilot and feasibility studies focused on evaluating acceptability and key uncertainties of an intervention [42]. For this feasibility trial, we aim to recruit 60 participants and 60 caretakers across multiple iterations of the Life Coping Program. While not based on a formal power calculation, the target sample reflects a realistic estimate based on anticipated recruitment capacity, available resources, and logistical considerations in the project. The chosen sample size is, due to the variety of primary conditions, slightly above comparable studies [38, 43], and will support estimation of recruitment rates, retention rates, and progression criteria with reasonable precision.

### Progression criteria

Following recommendations by El-Kotob et al. [44], a set of a priori criteria for progressing to a large-scale RCT has been established. High feasibility is defined as completion rate > 80%, moderate feasibility as 51–80%, and low feasibility as < 50%. When including 60 participants, the 95% Wilson score interval for the completion progression threshold of 80% will be 68% to 88% (calculated by R version 4.4.1). If the total completion rate shows high feasibility, we might conclude a large-scale RCT as feasible. If the total completion rate shows moderate feasibility, further investigation of collected data is warranted to evaluate whether modification to the intervention might be required. If the total completion rate shows low feasibility, we consider the evidence insufficient to justify proceeding to the definitive RCT. In such cases, modifications to the intervention would be required, followed by further trials until the predefined feasibility criteria are met.

### Risks

Potential harms of being included in the intervention and the assessments will be explicitly outlined in the participant explanatory statements and consent form. During the intervention, the children and adolescents are expected to engage in physical activity with varying degrees of activity intensity. During such activities, the participants can potentially injure themselves or others physically, and/or they can experience some physical or psychological distress. All activities required in the intervention are preplanned and designed to ensure maximum safety; however, any group-based physical activities inherently involve some risk of injury. All accidents and injuries will be recorded and reported to the Principal Investigator. In addition, any adverse events observed among the participants will be monitored routinely throughout the study and be presented in later publication. Any such adverse events will be immediately reported back to the responsible referring specialist. All study therapists are experienced in working with children and adolescents.

## Discussion

This study will explore the feasibility of a group-based intervention designed to enhance HRQoL and physical activity among children and adolescents with chronic health conditions. The overarching goal is to establish an evidence base for this new clinical intervention, which may improve outcomes and reduce future healthcare utilization in this population.

We anticipate that participation in the Life Coping Program will lead to improvements in HRQoL and increased levels of physical activity. Regarding measures of feasibility, we expect to achieve satisfactory estimates for recruitment, fidelity, completion, and retention. Additionally, we anticipate that results from the secondary outcomes will provide a basis for effect size estimation for future effect studies. Finally, we expect that both children, adolescents, and caregivers will find the intervention acceptable.

However, due to limited resources and the intervention already being ongoing, the trial is limited in several aspects. No interviews or cost–benefit and consequence analyses are included in the trial. Further, only limited parts of output and short-term outcomes in the logic model (Fig. 2) are evaluated in this trial.

One of the study’s strengths is its alignment with the MRC framework for evaluating complex health interventions [34]. The findings from this research will be pivotal in advancing the subsequent stages of the MRC framework, particularly in the development and implementation of a definitive randomized controlled trial assessing the effectiveness of the Life Coping Program in improving HRQoL and physical activity levels for children and adolescents with chronic illnesses or medical conditions.

Moreover, the target population for this intervention consists of children and adolescents, who are particularly at risk of experiencing low HRQoL and insufficient levels of physical activity. Consequently, addressing these issues within this demographic can lead to substantial improvements in both short-term well-being and long-term health outcomes**.**

## Trial status

The study is currently ongoing with participant inclusion having started in May 2023, and data collection is ongoing throughout the first quarter of 2025. Results from the study will be submitted for publication in the third quarter of 2025.

## Data Availability

The treatment manual for Life Coping Program is available on request (in Norwegian). Data sharing is not applicable to this article as no datasets were generated or analyzed.

## Appendix

SPIRIT 2025 checklist for feasibility trials, Spirit table, Flow Chart, Checklist for health personnel (Norwegian), Self-reported activity levels and satisfaction with relevant activities (Norwegian), Parents’ perception of their child’s activity levels and satisfaction with relevant activities (Norwegian), Evaluation form for the Life Coping Course (child or adolescent, Norwegian) and Evaluation form for the Life Coping Course (caretaker, Norwegian).
